# Farmers’ perceptions on tomato early blight, fungicide use factors and awareness of fungicide resistance: Insights from a field survey in Kenya

**DOI:** 10.1371/journal.pone.0269035

**Published:** 2023-01-23

**Authors:** Andrew M. Nuwamanya, Steven Runo, Maina Mwangi

**Affiliations:** 1 Department of Agricultural Science and Technology, School of Agriculture and Enterprise Development, Kenyatta University, Nairobi, Kenya; 2 Department of Biochemistry, Biotechnology and Microbiology, School of Pure and Applied Sciences, Kenyatta University, Nairobi, Kenya; Ghazi University, PAKISTAN

## Abstract

Early blight (EB) caused by *Alternaria solani* is one of the most devastating tomato diseases in Kenya and is most often managed by application of synthetic fungicides. However, there have been reports from farmers about the declining efficacy of some fungicides. These reports suggest that *A*. *solani* populations in Kenya could be developing resistance to some of the commonly used fungicides. In this study, we surveyed 175 tomato fields, sampled in 3 major tomato producing counties in Kenya, to determine the status of EB, management practices, and fungicide use factors that could contribute to development of resistance to fungicides among *A*. *solani* populations in Kenya. Data was recorded on farm characteristics, EB prevalence, fungicide usage, and farmers’ perceptions on fungicide efficacy. EB was prevalent in 85% of the fields and 90% of the farmers identified it as a major cause of yield loss. Tomato was grown all year round on 60% of the fields with only short fallow periods. All farmers reported that they were relying on fungicides for EB control and none among the cultivars grown was resistant to the disease. A total of 40 fungicide products, representing 20 active compounds with varying FRAC resistance risk levels were in use against EB. Majority (83%) of the farmers were applying fungicides at dosages and frequencies higher than those indicated on labels. Most farmers (81%) indicated that they had observed declines in effectiveness of at least one fungicide, used at EB control. This observation was more with fungicides in the strobilurin and triazole groups. These findings demonstrate that the current tomato production systems in Kenya do not take into account the risk of *A*. *solani* developing resistance to fungicides. Enhancing farmers’ knowledge of the disease and their ability to properly select and apply fungicides is therefore crucial for effective control of EB and mitigating the high risk of fungicide resistance build up.

## 1. Introduction

Tomato (*Solanum lycopersicum* L.), is among the world’s most important crops in terms of production, consumption, and trade. It belongs to Solanaceae family with origins in Central and South America [1]. In Sub-Saharan Africa (SSA), tomato is extensively grown as a food and cash crop, and contributes significantly to nutrition, employment, and income generation [2, 3]. According to [2], Kenya is among the leading tomato-producing countries in SSA, with a production of 599,458 tonnes. The crop accounts for about 7% of horticulture and 14% of vegetable production in the country [4].

Despite the importance of the crop, tomato yields in Kenya have been declining due to a number of biotic and abiotic constraints. Early blight (EB) caused by *Alternaria solani* (Ellis and G. Martin) Sorauer is among the most significant tomato diseases in Kenya [5, 6]. Early blight and Late blight (*Phytophthora infestans*) together, are estimated to cause 95.8% of all pre-harvest tomato yield losses in Kenya [7]. Aerial parts of plants infected by EB develop brownish black lesions that expand, causing plants to lose more and more of their photosynthetic tissue [5], ultimately producing smaller and lesioned fruits that fetch a low market value.

Management of early blight has remained a challenge, especially among smallholder farmers in Kenya [8]. In the past, there has been an increasing tendency by Kenyan farmers to rely on fungicides to control the disease, mostly because of their high control efficacy. According to [7], the highest pesticide use during tomato production in Kenya is for control of early and late blights with up to 40 applications per cropping season. The approved fungicides for EB control in Kenya include multisite actors, quinone outside inhibitors, demethylation inhibitors and Succinate dehydrogenase inhibitors [9]. However, there has been a growing concern from farmers about the declining efficacy of some of the fungicides at controlling early blight [10, 11], and this is complicating EB management.

In other tomato growing countries where such fungicide efficacy declines have been experienced, research usually confirms development of resistance in *A*. *solani* isolates to available fungicides [12–14]. Such resistance culminates from an interplay of factors such as agronomic practices, fungicide application techniques, pathogen genetics, and climate change [15, 16]. To prevent/delay fungicide resistance, early detection and deployment of counter strategies such as fungicide rotations, and alternative disease management strategies is necessary [17, 18].

However, in Kenya, no studies have been carried out to establish the current status of EB in fields and its chemical control despite many farmers’ complaints about the increasing EB severity and decline in efficacy of some fungicides used for its control [10, 11]. Consequently, Pesticide Regulatory agencies and fungicide distributors often attribute the low fungicide efficacy to misuse by farmers, while scientists speculate resistance development. However, there is no empirical evidence to support either of these hypotheses and hence recommendations for managing fungicide resistance often suffer from this knowledge gap.

Therefore, the objective of this study was to determine the status of tomato early blight and management practices in three major tomato growing regions in Kenya; and to assess the factors that could potentially contribute to development of fungicide resistance among the country’s *A*. *solani* populations. The factors considered are those that have been used in developing fungicide resistance risk assessments for other major crop diseases [15, 16, 19], and included field characteristics, fungicide chemistry, and application practices.

## 2. Materials and methods

### 2.1 Study sites

**Fig 1:**
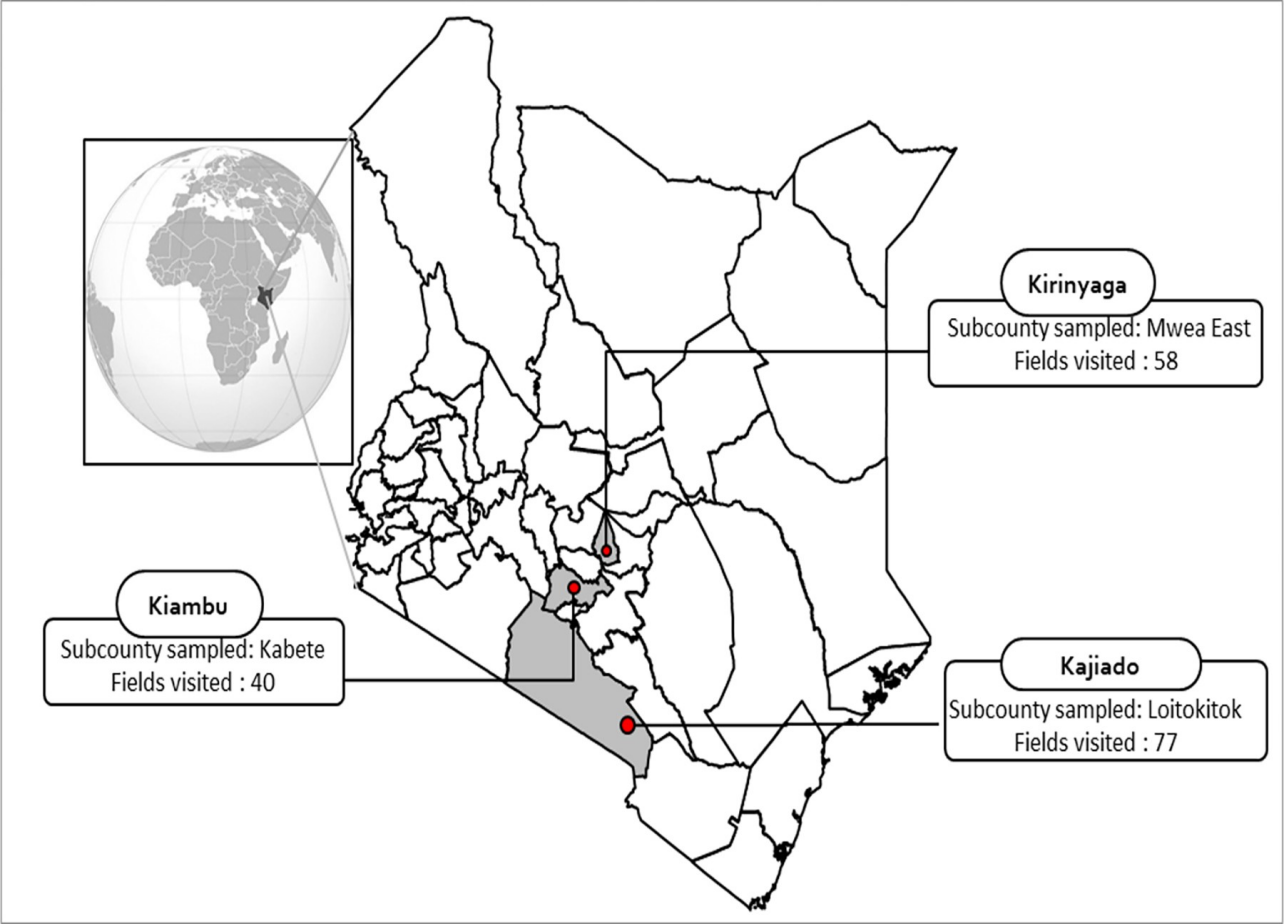
Map of Kenya showing location of study sites. (Developed for illustration purpose at http://yourfreetemplates.com).

This study was conducted in three major tomato growing areas in Kenya, representing three counties in Central and Rift valley regions (Fig 1). These were: Mwea East in Kirinyaga County, Kabete in Kiambu County, and Loitokitok in Kajiado County.

Mwea East is classified as a humid agroclimatic zone [20]. Located at the foothills of Mt. Kenya in the central region, the area receives an average annual rainfall between 1212 – 2146mm while average annual temperature ranges between 8–30°C [21]. Coupled with fertile vertisols in most areas, such conditions enable production of a wide variety of crops all year round, including tomato [22]. However, the warm humid conditions in the area are conducive for many fungal pathogens including *A*. *solani* that causes early blight [23–25].

Kabete is another significant tomato-growing hotspot in Central Kenya. Over the years, many greenhouses have been erected in the area [26], and produce a variety of short maturing horticultural crops for the rich market in the neighboring Nairobi metropolitan. This part of Kiambu County has been classified as a semi-humid ACZ [27]. Average annual rainfall in the area ranges between 600-2000mm while temperature averages between 18–22°C.

Loitokitok sub-county in Kajiado county lies on the foothills of Mt. Kilimanjaro in the southern region of Kenya, bordering Tanzania. Average annual rainfall ranges between 475 – 750mm while temperature is between 12–27°C [21]. Classified as a semi-arid ACZ [27], the sub-county consists of small areas with water availability surrounded by expansive dry grasslands. Tomato production here is dominated by small-scale farmers who must irrigate their fields all year round. The warm conditions in the sub-county also favor a wide range of tomato diseases including Early blight.

### 2.2 Sampling procedure

The number of tomato fields to study in each county was obtained using Yamane’s Formula for sample size [28] (Eq 1).


$$n=\frac{N}{{1+N(e)}^{2}}$$ (1)

Where,

n = sample size,

N = Number of tomato fields in a county (according to the County Agriculture Office),

e = level of precision or sampling error (5%).s

After determining the number of farms required in each area, fields were selected systematically along predetermined routes at 1 km intervals. In exceptional cases where there was no field available, the nearest tomato field was sampled. In total, 175 tomato fields were visited; 58 in Kirinyaga, 77 in Kajiado, and 40 in Kiambu.

### 2.3 Data collection

Data was collected through interviews with farmers (using a semi-structured questionnaire) and direct observation of tomato production practices. Details were captured about the farmer, e.g. age, gender, experience in tomato production; and about the farm, e.g. area under tomato, number of cropping cycles per year, varieties grown, irrigation methods, prevalent pests and diseases, and EB management practices including fungicide application procedures.

To assess the importance of early blight, respondents were asked to rate prevalent pests and diseases, in terms of yield loss caused. Here, a 1–4 scale developed for this study was used wherein; 1 represented low yield loss (<10%), 2-moderate (11–20%), 3-high (21%-30%), and 4 -very high yield loss (>40%). Respondents were also asked to state the control strategies used for EB control. Farmers who reported to be applying fungicides were asked to identify each product by its trade name, and to provide information on dosage and frequency of sprays. Farmers were also asked to rank the effectiveness of fungicides using a simple rating scale of low (1), moderate (2), and high (3).

Data was processed in Microsoft Excel version 2013 and analyzed in SPSS software version 23. The risk of resistance developing for the active compounds used, was determined using the Fungicide Resistance Action Committee (FRAC) classification available at *https*:*//www*.*frac*.*info*.

### 2.4 Ethics approval and consent to participate

Approval to conduct this research was obtained from National Council of Science, Technology and Innovation (NACOSTI), Kenya, License no. NACOSTI/P/21/13512. Verbal consent was obtained each participant before interview

## 3. Results

### 3.1 Demographic characteristics of tomato farmers

Males constituted the majority of farmers interviewed in the three study sites (Kirinyaga 84%, Kiambu 65%, and Kajiado 93%). Majority of farmers were aged 31–50 years (Kirinyaga 81%, Kiambu 92%, and Kajiado 87%). All farmers had attained some formal education. i.e Primary (39%), secondary (51%) and tertiary (18%). Farmers’ experience with tomato growing varied from 1 to 40 years, the average being 9.4 years (Table 1).

**Table 1:** Selected characteristics of interviewed tomato farmers in Kirinyaga, Kiambu, and Kajiado counties.

Characteristic	Kirinyaga (n = 58)	Kiambu (n = 40)	Kajiado (n = 77)	Overall (n = 175)
Gender (% respondents)				
Male	84.5 (49)	65.0 (26)	93.5 (72)	84.0 (147)
Female	15.5 (9)	35.0 (14)	6.5 (5)	16.0 (28)
Age (% respondents)				
20–30 years	6.9 (4)	2.5 (1)	9.1 (7)	6.8 (12)
31–40 years	44.8 (26)	37.5 (15)	58.4 (45)	49.1 (86)
41–50 years	36.2 (21)	55.0 (22)	28.6 (22)	37.1 (65)
>50 years	12.1 (7)	5.0 (2)	3.9 (3)	9 (12)
Formal education (% respondents)				
Primary	37.9 (22)	25.0 (10)	46.8 (36)	38.8 (68)
Secondary	44.8 (26)	72.5 (29)	44.1 (34)	50.9 (89)
Tertiary	17.2 (10)	2.5 (01)	9.1 (7)	10.3 (18)
Av. tomato growing experience (years± SD*)	10.4±5.2^a^	7.4±2.3^b^	9.6±4.8^c^	9.4±4.1

Figures in parentheses represent frequencies of the responses.

*Standard deviation. Letters indicate significant differences between means at α = 0.05

### 3.2 Characteristics of the tomato fields

Individual farm characteristics varied significantly (P < 0.05) across counties (Table 2). Kajiado had the highest average tomato acreage (1.9 ha) followed by Kirinyaga (1.5 ha) and lastly, Kiambu (0.6 ha). On majority (81%) of the farms, tomato was grown on open fields. Greenhouses were only common in Kiambu accounting for 80% of all tomato farms visited. The average estimated tomato yield was highest in Kiambu (11.7 tons/ha) and lowest in Kajiado (6.9tons/ha). Farmers’ estimated yield was significantly higher (P = 0.041) under greenhouse production than in open fields.

**Table 2:** Characteristics of tomato fields.

Characteristics	Kirinyaga	Kiambu	Kajiado	Overall
	(n = 58)	(n = 40)	(n = 77)	n = 175
Av. farm size/ha ±SD	2.0±3.2b	1.0±0.4ab	2.3±1.9a	1.75±2.5
Av. tomato acreage /ha±SD	1.5±2.2ab	0.6±0.2b	1.9±1.1a	1.33±1.7
Estimated tomato yield* (ton/ha±SD)	7.1±1.6ab	11.7±2.7a	6.9±4.5b	8.57±2.8
Production system (% fields)				
Green house	1.7 (1)	80.0 (32)	0.0	18.2 (32)
Open field	98.3 (57)	20.0 (8)	100.0 (77)	81.1 (142)
Cropping pattern (% fields)				
Monocrop	63.7 (37)	77.5 (31)	75.3 (58)	72 (126)
Intercrop	36.2 (21)	22.5 (9)	24.7 (19)	28 (49)
Irrigation method (% fields)				
Drip	1.7 (1)	75.0 (30)	3.9 (3)	19.4 (34)
Furrow	93.1 (54)	12.5 (5)	84.4 (65)	70.8 (124)
Sprinkler	0.0	12.5 (5)	6.5 (5)	5.8 (10)
Others (watering can, diversion channels)	5.2 (3)	0.0	5.2 (4)	4.0 (7)

*Farmers could estimate yield in terms of number of ‘crates’ or ‘Forwards’ harvested. Crates are wooden square containers with a capacity of 60-80Kg. ‘Forwards’ are trucks used to transport tomatoes to the market and each could carry an estimated 2 tonnes of tomatoes. *P-value at α = 0.05. SD- Standard deviation. Letters indicate significant differences in variables between study sites. Figures in parentheses indicate frequencies of categorical variables.

Tomato was grown as a monocrop in majority of the fields. Intercropping was only practiced on 28% of the farms with common intercrops being maize, beans, and green pepper. All farms visited practiced some form of irrigation. Majority of farmers (71%) used furrow, 19% drip, and 9% sprinkler irrigation. In Kirinyaga and Kajiado, other forms of irrigation were used e.g. watering cans and diversion channels on fields neighboring streams. These were observed on 4% of all the fields visited.

### 3.3 Prevalence of Early blight and other biotic constraints

A total of five major tomato diseases and four insect pests were present on at least 20% of the surveyed fields (Table 3). Early blight was the most prevalent disease (85% of fields), followed by late blight (83%). Most farmers could identify EB as *“Baridi”* (Swahili word for cold), an indication that they associated it with cold weather. Blights (early and late) were also the highest-ranked diseases in terms of yield loss caused. Early blight prevalence and overall yield loss rank were significantly highest (P<0.05) in Kirinyaga and lowest in Kiambu.

**Table 3:** Prevalence and farmer’s ranking of major biotic constraints to tomato production.

		Kirinyaga			Kiambu		Kajiado		Overall	
				Rank^a^		Rank^a^		Rank^a^		Rank^a^
Diseases^b^	Scientific name	% fields	(x±SEM)		% fields	(x±SEM)	% fields	(x±SEM)	% fields	(x±SEM)
Early blight	*Alternaria solani*	91.4	3.5± 0.09		75.0	2.4 ±0.16	90.9	3.1±0.09	85.2	3.1±0.08
Late blight	*Phytophthora infestans*	87.9	3.3± 0.08		68.5	2.2± 0.14	80.5	3.5±0.06	80.6	3.2±0.06
Bacterial wilt	*Ralstonia solanacearum*	60.3	1.2± 0.06		94.3	2.1± 0.15	22.1	1.5±0.20	48.0	1.3±0.06
Fungal wilts	*Fusarium spp*.,*Verticillum spp*,	19.0	1.2± 0.12		25.7	1.1± 0.07	19.5	1.4±0.13	20.6	1.5±0.13
Viral diseases	TSWV, TCMV, TYLCV	34.5	1.2± 0.12		34.2	1.3± 0.09	40.3	1.8±0.07	37.1	1.4±0.06
Pests^b^										
Leaf miners	*Tuta absoluta*	93.1	2.5± 0.16		94.3	3.1 ±0.12	93.5	3.9±0.04	89.8	3.1±0.07
Thrips	*Thrips tabaci*	87.9	2.2± 0.12		60.0	1.8 ±0.11	28.6	1.2±0.17	53.1	1.8±0.08
Spider mites	*Tetranychus evansi*	58.6	2.4± 0.15		40.0	1.1 ±0.06	62.3	1.4±0.11	54.2	1.4±0.12
Whiteflies	*Bemisia tabaci*	51.7	2.4± 0.07		80.0	1.8± 0.09	53.2	2.2±0.12	55.9	1.5±0.07

^a^ In each farm, each disease and pest was ranked by the farmer, based on estimated yield loss caused, using a 1–4 scale where 1 low (<10%), 2 Moderate (20–29%), 3 High (30–40%), 4 Very high (>40%).

^b^ Multiple answers possible

Other major diseases identified in the fields included Bacterial wilt (48%), Fungal wilts (21%), and viral diseases (37%) (Table 3). The observed viral disease symptoms resembled those of Tomato Common Mosaic Virus (TCMV), Tomato Spotted Wilt Virus (TSWV), and Tomato Yellow Leaf Curl Virus (TYLCV). The major pests in the fields were tomato leaf miners (90%), thrips (53%), spider mites (54%), and whiteflies (56%). Blights and tomato leaf miners were the highest-ranked biotic constraints (average overall ranks above 3).

### 3.4 Tomato varieties cultivated

**Fig 2:**
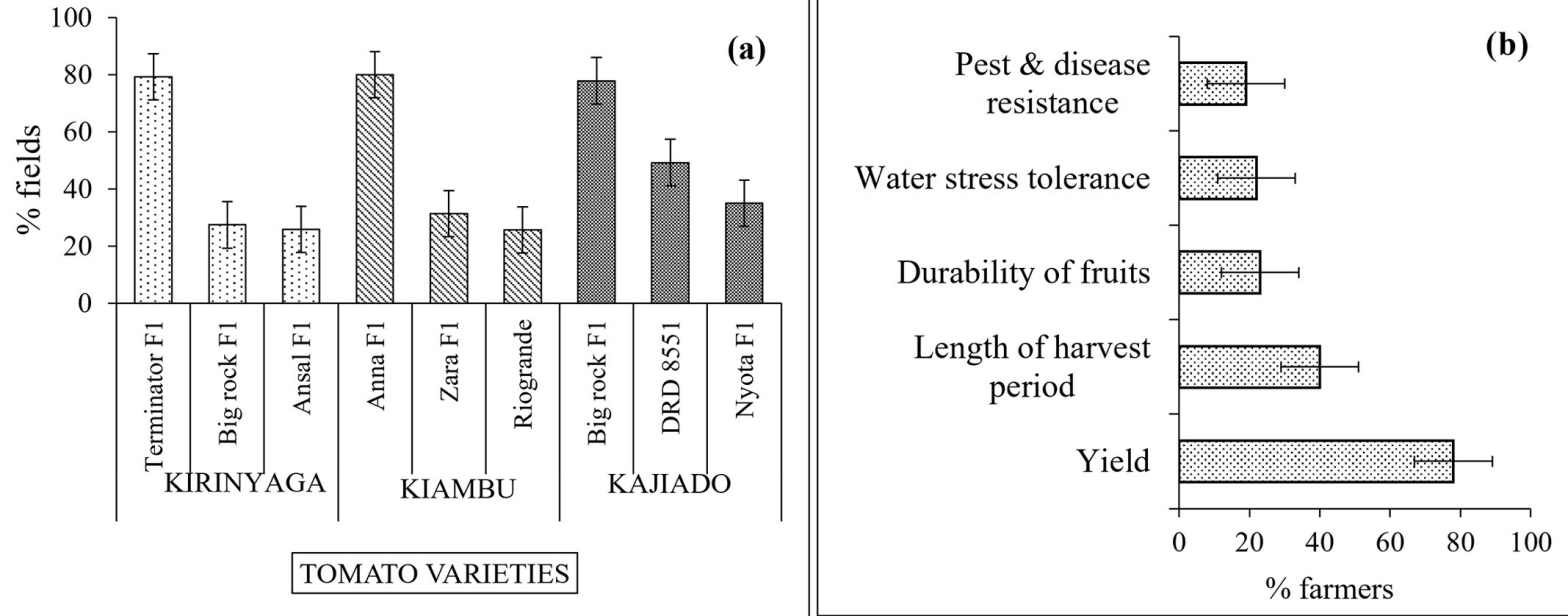
(a) Major tomato varieties in each county (b) Major factors influencing farmers’ choice of varieties.

A total of 19 tomato varieties were being grown in the different regions surveyed. A total of 45% of the farmers grew one variety at a time while the rest grew more than one variety. Cultivars, Anna F1 and Zara F1 were popular in Kiambu while Big rock F1 and DRD F1 were dominant in Kajiado. The most popular varieties in Kirinyaga were Terminator F1, Big rock F1, and Ansal F1 (Fig 2A). Whereas all these are improved varieties, their resistance/susceptibility to Early blight could not be ascertained as this information was not found on their seed packs and neither was it available in any literature.

Among the reasons for choice of cultivars, yield (78%) was the most mentioned (Fig 2B). Other factors included longevity of harvesting period (40%), size of fruits (23%), shelf life of fruits (22%), drought tolerance (16%), and price of seedlings (15%). Resistance of the selected variety to pests and diseases was only considered important by 19% of the farmers interviewed. The seedlings on most fields were purchased from commercial nurseries; Only 4 farmers (representing 2% of the total) propagated their own seedlings.

Majority (66%) of the farmers could grow tomatoes for more than two cropping cycles per year. Considering that the average tomato season in Kenya is 4–5 months, this means that many farmers in surveyed areas could plant a new crop in the same field within the same month, after harvesting the old one. Some fields had been under continuous tomato production for more than 10 years without any fallow periods or rotation with any other crop. Since all farmers practiced some form of irrigation, tomato production could be undertaken all year round without defined periods for planting or harvest.

### 3.5 Control methods used against Early blight

All farmers interviewed had been relying on synthetic fungicides for management of Early blight. Although cultural practices that can enhance EB control (e.g weeding, pruning, and staking) were observed on most farms, only 10% of the farmers could associate these with EB management. Biological control using fungal antagonists (under experimental trial) were observed on only two farms, one in Kiambu and one in Kirinyaga. Only 7% of the farmers interviewed were aware of integrated disease management approaches.

#### 3.5.1 Composition and resistance risk of fungicides used

A total of 40 fungicide products representing 20 active compounds were in use against Early Blight in the different counties (Table 4). Of these, 24 contained one active compound while 16 had mixtures of active ingredients. Active compounds represented 6 chemical groups with different modes of action. Mancozeb (present in 15 out of 40 fungicides) was the most common active compound. Others were propineb, cymoxanil, chlorothalonil, triazoles, carbendazim, and azoxystrobin. Most active compounds (14 out of 20) fell in FRAC resistance risk categories above ‘Low’ (Table 4).

**Table 4:** Active ingredients in fungicide products used to control tomato early blight and their FRAC resistance risk codes.

Fungicide Trade name	Active compound(s)	FRAC Resistance risk* (Code/meaning)
Ridomil Gold®	Metalaxyl + Mancozeb	(4/High) + (M3/Low)
Milraz®	Propineb + cymoxanil	(M3/Low) + (27/Low to medium)
Oshothane®	Mancozeb	M3/Low
Mistress®	Cymoxanil+ Mancozeb	(27/Low to Medium) + (M3/Low)
Agromax®	Cymoxanil+ Mancozeb	(27/Low to Medium) + (M3/Low)
Ortiva®	Azoxystrobin	11 (High)
Milthane Super®	Mancozeb	M3/Low)
Antracol®	Propineb	M3/Low)
Victory®	Metalaxyl+ Mancozeb	(4/High) + (M3/Low)
Score®	Difenoconazole	3 (Medium)
Linkmil®	Mancozeb+ Metalaxyl	(4/High) + (M3/Low)
Bayfidan®	Triadimenol	3/Medium
Classic®	Tebuconazole	3 /Medium
Daconil®	Chlorothalonil	M5 /Low
Funguran®	Copper hydroxide	M1/Low
Wetsulf®	Sulfur	M2/Low
Goldazim®	Carbendazim	1/High
Cover®	Azoxystrobin+ Propiconazole	(11/High) + (3/Medium)
Isacop®	Copper oxychloride	M1/Low
Greencop®	Copper oxychloride	M1/Low
Blue Shield®	Copper hydroxide	M1/Low
Ivory®	Mancozeb	M3/Low
Penncozeb®	Mancozeb	M3/Low
Equation Pro®	Cymoxanil	M3/Low
Bayleton®	Femoxadone	Unclassified
Komesha®	Cymoxanil+ Propineb	(27/Low to medium) + (M3/Low)
Absolute®	Azoxystrobin + Difenoconazole + Hexaconazole	(11/High) + (3/Medium) + (3/Medium)
Rodazim®	Carbendazim	1/High
Trinity Gold®	Copper oxychloride+ Cymoxanil+ Mancozeb	(M1/Low) + (27/Low) + (M3/Low)
Nordox®	Copper	M1/Low
Volar MZ®	Dimethomorph + Mancozeb	(40/Low to medium) + (M3/Low)
Azoxystop®	Azoxystrobin + Difenoconazole	(11/High) + (3/Medium)
Nativo®	Trifloxystrobin + Tebuconazole	(11/High) + (3/Medium)
Ranson®	Carbendazim+ Triadimefon	(1/High) + (3/Medium)
Mixanil®	Cymoxanil+ Chlorothalonil	(27/Low to Medium) + (M5/Low)
Farmerzeb®	Mancozeb	M3/Low
Z-Force®	Mancozeb	M3/Low
Stargem®	Mancozeb	M3/Low
Tajiri®	Mancozeb+ Cymoxanil	(M3/Low) + (27/ Low to Medium)
Top Guard®	Thiophanate methyl	1/High

*FRAC = Fungicide Resistance Action Committee. The risk codes were obtained online at https://www.frac.info

#### 3.5.2 Farmers’ preference for fungicide products

Farmers’ preference for fungicide products differed significantly across the study sites (Table 5). The most commonly used brand names were Ridomil Gold® (60%), Milraz® (29%), and Oshothane® (21% of fields). Price of the fungicide (72%), prevailing weather (70%), and perception of efficacy (67%) were the major factors influencing the choice of fungicides. With exception of Ridomil Gold®, the effectiveness ranking of fungicide products did not differ significantly (at α = 0.05) across study sites. Ridomil Gold® (overall rank 2.1) and Milraz® (2.2) were ranked as the most effective. Coincidentally, these were also the most expensive.

**Table 5:** Fungicide products used to control tomato early blight in three surveyed counties of Kenya, January-March 2021.

	Kirinyaga		Kiambu		Kajiado		Overall			
Brand name^a^	% fields	Rank ^b^ (x±SEM)	% fields	Rank^b^ (x±SEM)	% fields	Rank ^b^ (x±SEM)	% fields	Rank ^b^ (x±SEM)	P value^c^	P value^d^
Ridomil Gold®	81.0	2.5± 0.09	94.3	2.4 ±0.16	41.6	2.2± 0.14	66.2	2.1±0.08	0.044	0.048
Milraz®	55.2	2.3± 0.08	42.9	2.2± 0.14	3.9	2.1± 0.15	28.5	2.2±0.06	0.039	0.052
Oshothane®	31.0	2.2± 0.06	40.0	2.1± 0.15	5.2	1.1± 0.07	20.5	1.8±0.06	0.043	0.061
Mistress®	32.8	1.2± 0.12	31.4	1.1± 0.07	3.9	1.3± 0.09	20.0	1.5±0.13	0.048	0.068
Agromax®	10.3	1.2± 0.12	5.7	1.3± 0.09	29.9	1.5± 0.16	17.7	1.4±0.06	0.043	0.087
Milthane Super®	8.6	1.1± 0.16	20.0	1.2± 0.09	6.5	2.2± 0.12	10.3	1.1±0.13	0.047	0.075
Antracol®	6.9	1.2± 0.12	15.0	1.3± 0.09	18.8	1.5± 0.16	14.3	1.3±0.06	0.043	0.087
Ortiva®	8.6	1.0± 0.09	5.7	1.1± 0.19	25.0	1.0± 0.05	15.4	1.0±0.06	0.043	0.087
Score®	6.9	1.0± 0.13	20.0	1.2± 0.07	12.5	1.3± 0.17	12.0	1.1±0.13	0.047	0.075

^a^Multiple answers allowed.

^b^Farmers’ ranked the effectiveness of each fungicide using a 1–3 rating scale where 1 = low, 2 = Moderate, 3 = High

^c^P value for preference of fungicide products at α = 0.05

^d^P value for fungicide ranking at α = 0.05

According to most farmers, Early blight became severer during cold periods. During such times, fungicide products perceived to be the most effective (i.e Ridomil Gold® and Milraz®) were mostly applied. Most farmers interviewed (81%) viewed chemical control of Early blight using available fungicides as moderately effective, only 19% ranked it as highly effective.

#### 3.5.3 Fungicide dosages and spray interval

**Fig 3:**
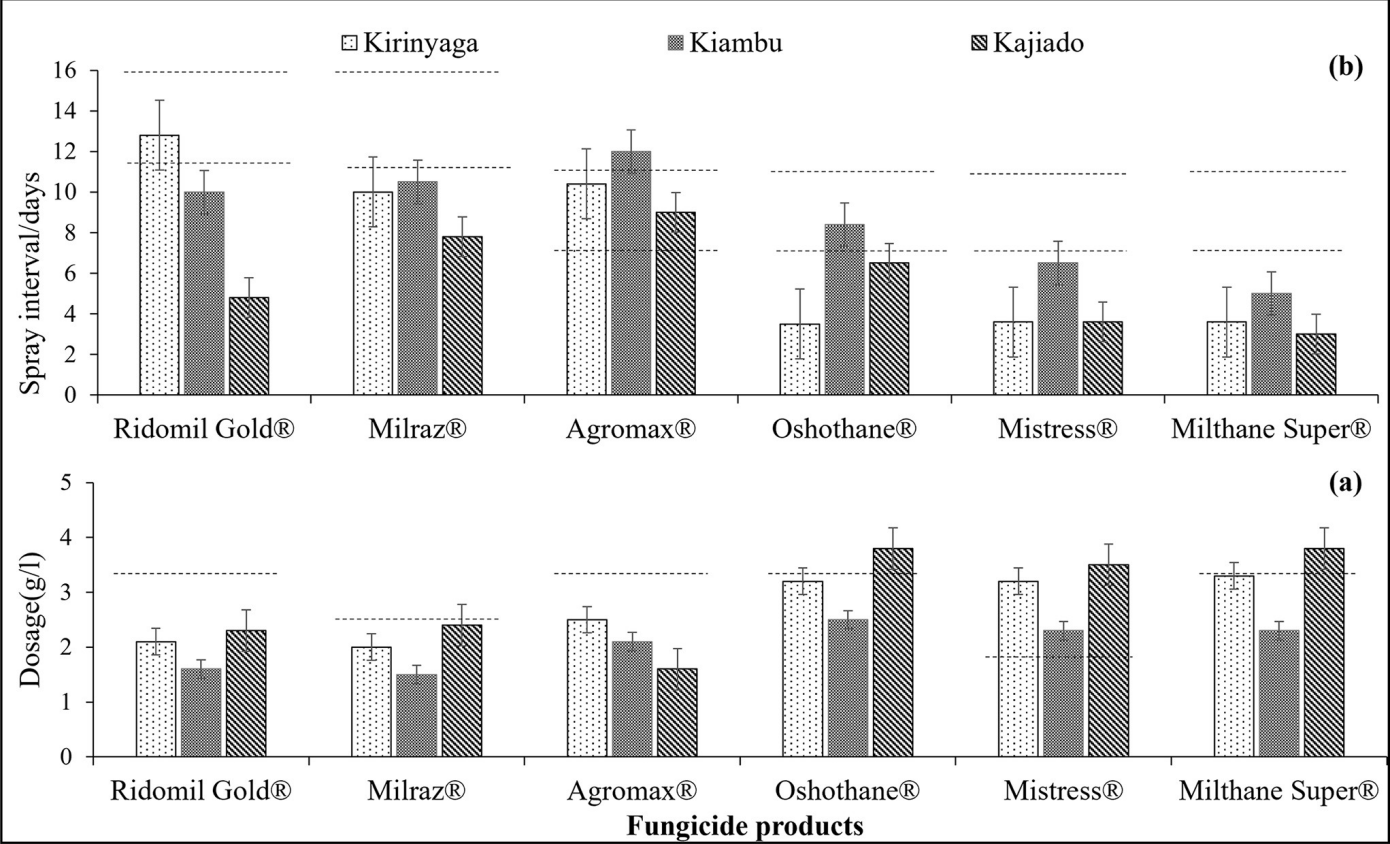
Farmer’s application rates for 6 most commonly used fungicide products against Early blight relative to manufacturer’s recommended levels (indicated by dotted lines) (a) Average farmers’ spray dosages (b) Average farmers’ spray intervals. *Farmers’ dosages were calculated from responses on volume of fungicide per Knapsack pump(16l/20l) or mixing drum (320l/800l)*. ^*c*^*Farmer’s spray intervals were calculated from responses on number of sprays per week or month*.

On majority of the fields (83%), farmers did not follow the manufacturers’ recommendations on fungicide dosage and spray intervals. Most applied higher than recommended doses especially of the low cost fungicides, in an attempt to increase their effectiveness (Fig 3).

Price (90%), weather (74%), and perceived effectiveness (67%) were the major factors influencing spray dosage and application intervals. Higher doses were applied during cold periods than on warm ones. The average dosage for the more costly fungicides (i.e Ridomil Gold® and Milraz®) were lower than those recommended by the manufacturers while the dosage for the less expensive products tended to be much higher. The spray intervals were also shorter for less costly fungicides than compared to more expensive ones. Comparatively, the fungicide dosage was lowest and spray intervals longest in Kiambu. Kajiado had the highest fungicide dosages and shortest spray intervals.

#### 3.5.4 Timing of fungicide application

On most fields in Kirinyaga (91%) and Kajiado (78%), fungicide application usually commenced in the first week after transplanting (Table 6). In Kiambu, where most production took place in greenhouses, the first fungicide application would occur much later. Majority of farmers (74%) indicated that they applied fungicides as a preventive measure for early blight. (Table 6).

**Table 6:** Decision factors on fungicide application among tomato farmers in Kirinyaga, Kiambu, and Kajiado counties of Kenya, January-March 2021.

	Kirinyaga	Kiambu	Kajiado	Overall
What informs your decision to start applying fungicides? %				
Cold weather	-	20 (8)	-	4.6 (8)
First EB symptoms	17.2 (10)	50.0 (20)	10.4 (08)	21.7 (38)
EB Prevention	82.8 (48)	30.0 (12)	89.6 (69)	73.7 (129)
When after planting does the first fungicide application occur? %				
In the first week	91.3 (53)	12.5 (5)	77.9 (60)	67.4 (118)
In the first 2 weeks	8.6 (5)	25.0 (10)	22.1 (17)	18.2 (32)
In the first month	-	62.5 (25)	-	14.3 (25)
Factors for choice of fungicide* (% farmers)				
Price	72.4 (42)	65.7 (23)	75.3 (58)	72.4 (123)
Weather	86.2 (50)	54.2 (19)	64.9 (50)	70.0 (119)
Effectiveness	63.7 (37)	71.4 (25)	67.5 (52)	67.0 (113)

*Multiple answers allowed. *Figures in parentheses represent frequencies of the responses*

#### 3.5.5 Farmers’ perceptions on declining efficacy of fungicides

**Fig 4:**
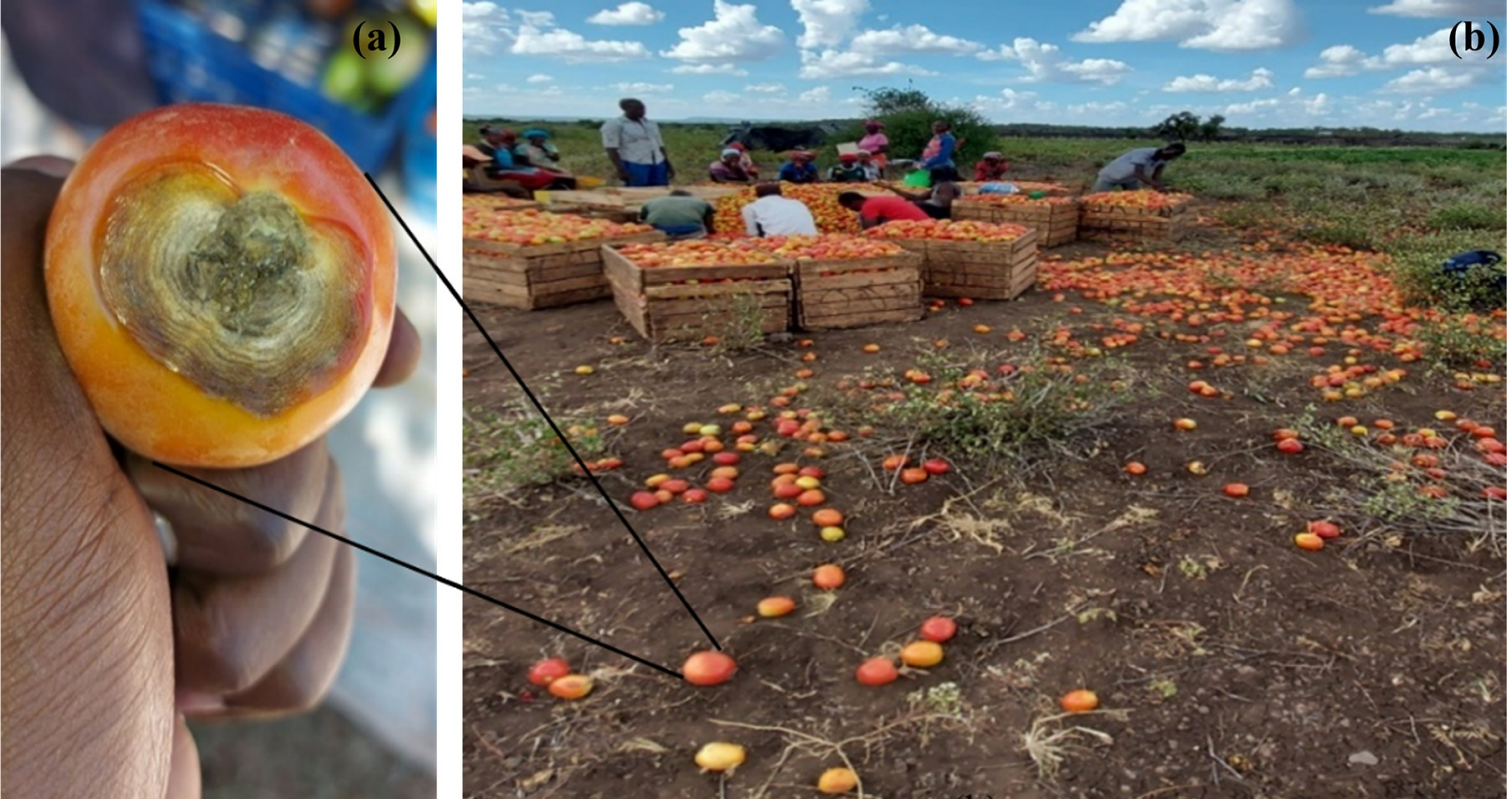
Typical early blight damage on a tomato fruit despite presence of fungicide residues (a) and large quantities of discarded damaged fruits (b). Approximately 30% of all harvested fruits were being discarded at this grading site in Gathigiriri village, Kirinyaga, County, Kenya (b).

Most farmers (81%) had observed declining efficacy of at least one fungicide product (Table 7). Kajiado county had the highest proportion of such farmers (92%), followed by Kirinyaga (88%) and lastly Kiambu (50%). To a typical farmer, the effect of such efficacy declines would be felt most, during fruit grading, whereby a large number of fruits (bearing EB lesions) would be discarded, even when fungicides were applied in the fields (Fig 4). A total of 25 fungicide products were perceived by farmers to have declined in efficacy against early blight. Such products contained mostly single-site active compounds e.g Azoxystrobin (60% of the mentioned products), difenoconazole (20%), and tebuconazole (20%). Farmers attributed the declining efficacy of fungicides to possible development of resistance by the pathogen (71% of the farmers), counterfeit products (31%), and climate change (19%).

**Table 7:** Farmers’ perceptions on declining efficacy of some fungicides for Early blight control in surveyed counties, Kenya.

Farmers who had observed declines in efficacy of at least one fungicide (% farmers)	
Kirinyaga	87.9 (51)
Kiambu	50.0 (20)
Kajiado	92.2 (71)
Major a.i s in fungicide products perceived as having declined in efficacy against EB^a b^ (% products)	
Azoxystrobin	60 (15)
Tebunoconazole	20 (5)
Difenoconazole	24 (6)
Trifloxystrobin	20 (5)
Farmers’ reasons for declining efficacy of fungicides^b^ (% farmers)	
Resistance by the pathogen	71.4 (125)
Counterfeit products	31.4 (55)
Climate change	18.8 (33)
Didn’t know	24.0(42)

a.i–Active ingredient. a-Some mentioned fungicides contained more than one active ingredient. b-Multiple answers allowed. *Figures in parentheses represent frequencies of the responses*

## 4. Discussion

The key to effective management of plant diseases lies in regular monitoring of components of the disease triangle which is itself, a delicate balance between pathogen parameters, environmental conditions, and host plant resistance/susceptibility [29, 30]. In this era of climate change, plant pathogens are evolving rapidly, overcoming traditional crop protection options e.g by overcoming host resistance and/or developing resistance to chemical control options available to farmers globally [31, 32]. To our knowledge, this study has been the first attempt at establishing the status of Early blight in Kenya’s tomato fields, control methods used, and farmers’ perceived efficacy of available fungicides in Kenya.

### 4.1 Demographic characteristics of farmers interviewed

The results indicate that males constituted the majority of tomato farmers across the three counties under study. This is consistent with findings from related studies done in Kenya [4, 22, 33, 34]. The male dominance has been attributed to the fact that more males than females own and control access to key production factors (such as land and capital) in Kenya [4, 35, 36]. Therefore, since tomato production is a capital-intensive process, it was not surprising, that more males than females were involved in tomato growing in all the three studied counties.

Majority of the tomato farmers interviewed were aged between 31–50 years. This is consistent with findings from previous studies involving tomato farmers in Kenya [22, 37]. The fact that all farmers had attained some level of formal education is important since this has been associated with increased understanding of aspects of disease control at farm level [37, 38]. However, the findings suggest that there was inadequacy of knowledge on tomato diseases and their management, which underscores a need for regular training of farmers on up-to-date EB management strategies, regardless of their education level.

### 4.2 Characteristics of tomato fields in studied counties

The findings showed that respondents owned small farm sizes ranging from 1.0 ha in Kiambu to 2.3 ha in Kajiado. Land ownership patterns are important because land size limits application of important disease management practices for example crop rotation and fallowing [4]. As noted, tomato was grown for up to 3 times per year (without rotations) on majority of the surveyed fields. This practice provides conducive conditions for fungicide-resistant *A*. *solani* individuals to multiply quickly from innocula, transferred across successive seasons [39, 40], hence this presented a high resistance risk among *Alternaria solani* populations in surveyed areas.

### 4.3 Occurrence and farmers’ ranking of Early blight

The high yield loss associated with EB in Kirinyaga County can be attributed to the humid conditions experienced there, year-round [23–25]. In Kajiado (a warmer area), irrigation all year round (in combination with the warm climate) could have contributed to the high EB severity [41]. For Kiambu, the low yield loss may be due to dominance of greenhouse tomato production in the county which has been linked with low severity of EB [42, 43].

### 4.4 Management practices employed against Early blight

This study established that farmers in all the studied counties were relying on synthetic fungicides as the main method for EB control, confirming previous reports [4, 44]. In addition to this, Early blight-resistant tomato varieties were not yet available in surveyed areas; regardless of the yield losses, EB was causing in the fields. To increase the range of control options available, tomato breeders in Kenya, should consider incorporating EB resistance traits in the accessions being developed.

The observed cultural practices with potential to reduce EB severity provide a promising strategy to supplement use of fungicides. Majority of farmers planted certified, seedlings, most fields were weed-free and plant nutrition well managed. According to [15], such practices reduce the agronomic risk for resistance development and hence they should be promoted.

The active compounds in the most commonly used fungicide products against EB represented only six chemical groups and six modes of action. This is a low number of chemical groups compared to those registered for EB control in the country [11]. This may be attributed to dominance of a few products sold by multinationals with large marketing budgets. With *A*. *solani*’s proven ability to develop cross-resistance across fungicide classes [45–47], the narrow diversity of modes of action among the fungicides applied, presented a high resistance risk for the pathogen and could complicate the management of resistant strains in future. Sensitizing farmers about the need to increase the diversity of fungicides applied and rotating them regularly would help to address this challenge.

Most farmers indicated that they were not adhering to manufacturers’ recommendations on fungicide dosages and spray intervals. While this finding appeared to be associated with product cost and its perceived effectiveness, the study does not present any conclusive reasons and recommends this for further investigation. The high spray dosages in Kajiado may be attributed to high EB prevalence in the county, favored in part by the warm climate and also the fact that farmers could access fungicides more cheaply in neighboring Tanzania. Overdosing fungicides has been reported to favor establishment of fungicide-resistant individuals among pathogens [16, 48, 49].

### 4.5 Farmers’ perceptions on declining efficacy of fungicides

Majority of farmers interviewed had observed declines in performance of at least one fungicide, and could attribute this to possible resistance development by the pathogen. However, they were not employing any resistance management strategies, partly due to lack of awareness of counter-resistance practices such as fungicide alternations and IDM [18, 50].

Notably, the majority of fungicides that were reported to have declining efficacy against EB, contained active compounds with single-site modes of action (majorly strobilurins and azoles), which is coherent with literature [51–53]. According to [15], resistance develops faster for single-site fungicides since they target only one gene/stage in the fungal biochemical pathway. This means that even mutation of a single nucleotide is enough to modify the target site, making it difficult for the fungicide to effectively suppress pathogen populations.

## 5. Conclusion

The findings from this study demonstrate that current tomato farming systems and EB control practices in Kenya do not take into account, the high risk of *A*. *solani* developing resistance to fungicides. Therefore, there is a need to raise awareness of farmers about the high likelihood of fungicide resistance and build their capacity to implement effective mitigative measures.

## Supporting information

S1 Data (XLSX)

S1 Questionnaire (DOCX)
