# Supplementary material for: Farmers’ perceptions on tomato early blight, fungicide use factors and awareness of fungicide resistance: Insights from a field survey in Kenya
Source: PLoS One. 2023 Jan 23;18(1):e0269035. doi: 10.1371/journal.pone.0269035 (PMC9870120; doi:10.1371/journal.pone.0269035)
Supplement: S1 Questionnaire — (DOCX) [file pone.0269035.s002.docx]

# Survey questionnaire

I am a postgraduate student at Kenyatta University carrying out a survey to evaluate farmers' perceptions on Early blight and its management in tomato farms of Kajiado, Kiambu and Kirinyaga counties. You have been selected as one of the tomato farmers in this area. Information collected will only be used for academic and research purposes and will be treated with utmost confidentiality. In case of any question(s) concerning the study, Contact Andrew Nuwamanya (+254721381978) or email: [*amnuwamanya@gmail.com*](about:blank).

May I now request for your permission to begin the interview?

Yes No

Starting time:…………

End time: ……………..

| County | Subcounty | Location | Village | Farm ID. | GPS coordinates of the field |
| --- | --- | --- | --- | --- | --- |
|  |  |  |  |  |  |

**SECTION A: HOUSEHOLD CHARACTERISTICS**

1 (a) Name of the respondent

………………………………………………………....

(b)Mobile No

…………………………………………………………………………………

(c) Gender of the respondent: Male Female

(d) Age of the respondent (in years) ……………………………………………………………….

(e) What is the highest educational level attained by the respondent? None Primary Secondary Tertiary

**SECTION B: TOMATO PRODUCTION PRACTICES**

2 (a) How long have you been growing tomatoes? ............................................................................

(b)What is your total farm size? ……………………………………………………….................

(c) What is the size of your farm under tomato production (acres)? ……………

3 (a) Which varieties of tomato do you grow on your farm? Rank them in order of preference:

| Rank | Variety | Area size | Why chosen over others |
| --- | --- | --- | --- |
|  |  |  |  |
|  |  |  |  |
|  |  |  |  |
|  |  |  |  |

(b)(i) How many seasons do you grow tomato per year? ……….

(ii) In which months of the year do you have tomato in your field?...........……………………..

(c) What is the approximate tomato yield on your farm ? ………..

(d) (i) Which tomato production system do you use? Greenhouse…… Open field ……. Both….

(ii) If open field, what do you rely on as a source of water for your crop? Rainfall Irrigation Both

(iii) If you irrigate, which irrigation method do you use on your farm? Drip Furrow Sprinkler Others (Specify) …………

1. (a)(i) Which crops do you intercrop your tomatoes with?

…………….. ……………. ……………………………………………

(ii) Which crops do you grow adjacent to your tomato field?

…………… …………..

(b) How long do you wait to grow tomatoes again on your land after a season? ………….

**SECTION C: FARMERS’ KNOWLEDGE ON TOMATO PESTS AND DISEASES IN THEIR FARMS**

5. Which diseases and pests have been affecting your tomatoes? Rank them in terms of magnitude of yield loss caused Very high (> 40%)…… High (21-39%)…….Medium (11-20%)…… Low (1-10%)

(a) Diseases

| Rank | Disease | Estimated yield loss |
| --- | --- | --- |
| 1 |  |  |
| 2 |  |  |
| 3 |  |  |
| 4 |  |  |
| 5 |  |  |

(b) Pests

| Rank | Pest | Estimated yield loss |
| --- | --- | --- |
| 1 |  |  |
| 2 |  |  |
| 3 |  |  |
| 4 |  |  |
| 5 |  |  |

**SECTION C: MEASURES TAKEN BY TOMATO FARMERS TO MANAGE TOMATO DISEASES**

6. Which management practices do you use against diseases on your tomato farm? Rate their effectiveness by ticking where applicable

| **Management practice (Grouped into the following categories)** | **Effectiveness**  **High Moderate Low Not effective** | | | |
| --- | --- | --- | --- | --- |
| Cultural, e.g. early planting, intercropping, weeding, certified seed, mulching, crop rotation |  |  |  |  |
| Physical, e.g. hot water treatment of seeds, destruction of diseased crops |  |  |  |  |
| Biological e.g use of antagonistic bacteria and fungi |  |  |  |  |
| Chemical control through use of fungicides |  |  |  |  |
| Integrating measures? specify |  |  |  |  |
| Others (Specify) |  |  |  |  |

**7.** (a) For chemical control of early blights, when do you start applying the fungicide(s)? …………………………………………………………………………………

(b) What informs your decision to start applying the fungicide(s)?

……………………………………………………………………………………………………………………………………………………………………

(c) For each of the fungicide(s) used, State the trade name, dosage, frequency of spray and effectiveness rating (*H= Highly effective M= Moderately effective L= Less effective)*

| Fungicide trade name | Dosage | Frequency of spray: X1 /week; X1/ 2 weeks; X1 / month; X1/2months | Rate the Effectiveness of the fungicide H/ M/ L |
| --- | --- | --- | --- |
|  |  |  |  |
|  |  |  |  |
|  |  |  |  |
|  |  |  |  |
|  |  |  |  |

8. (a) Do you strictly follow dosage recommendations according to fungicide labels?

Yes No

(b) If No, State why not?

……………………………………………………………………………………………………………………………………………………………………

9. (a) Do you interchange between different fungicide chemical products in a tomato growing season?

Yes No

(b) (i) If Yes, is it always or some times?

Always Sometimes

(ii) State the reason(s) why you interchange between different chemical products in a growing season? …………………………………………………………………………………………

(c) (i) Have you observed any decrease in effectiveness of any fungicide over the years in your tomato growing? Yes No

(ii) If yes, state the brand names of fungicides whose effectiveness has decreased over the year(s).

……………………………………………………………………………………………………………………………………………………………….......................................................................................................................................................................................................................................................

(iii) Which factors do you think are responsible for the declining efficacy of some Early blight fungicides?

………………………………………………………………………………………………………………………………………………………………..............................................................................................................

10. What is your source of information regarding tomato production and pest management practices?

Radio……….. Tv………… Mobile phones……… Extension officer……….. Newspaper………..Others (Specify) …………..

**END***
